# Associations Between Carotid Plaque Characteristics and Perioperative Cerebral Blood Flow Determined by Arterial Spin Labeling Imaging in Patients With Moderate-to-Severe Stenosis Undergoing Carotid Endarterectomy

**DOI:** 10.3389/fneur.2022.899957

**Published:** 2022-07-05

**Authors:** Ying Liu, Ran Huo, Huimin Xu, Guangjin Zhou, Tao Wang, Huishu Yuan, Xihai Zhao

**Affiliations:** ^1^Department of Radiology, Peking University Third Hospital, Beijing, China; ^2^Department of Neurosurgery, Peking University Third Hospital, Beijing, China; ^3^Center for Biomedical Imaging Research, Department of Biomedical Engineering, School of Medicine, Tsinghua University, Beijing, China

**Keywords:** carotid atherosclerosis, magnetic resonance imaging, arterial spin labeling, cerebral blood flow, carotid endarterectomy

## Abstract

**Purpose:**

To examine the associations between carotid plaque characteristics and perioperative cerebral blood flow (CBF) by arterial spin labeling (ASL) imaging.

**Materials and Methods:**

Patients with unilateral moderate-to-severe carotid stenosis referred for carotid endarterectomy (CEA) were recruited and underwent carotid vessel wall and brain ASL magnetic resonance imaging. The following imaging features were measured: relative CBF (rCBF = CBF_index−*hemisphere*_/CBF_contralateral−*hemisphere*_) in the middle cerebral artery territory; plaque burden and the presence of lipid-rich necrotic core; intraplaque hemorrhage (IPH); calcification; ulcer and fibrous-cap rupture; and the volume and maximum plaque components' area percentages. The associations between plaque characteristics and perioperative CBF were analyzed.

**Results:**

Sixty-one patients (mean age, 66.6 ± 7.8 years; 55 males) were included. Univariate linear regression showed that rCBF_pre−CEA_ was associated with stenosis [β, −0.462; 95% confidence interval (CI), from −0.797 to −0.126; *p* = 0.008], calcification (β, 0.103; 95% CI, 0.005–0.201; *p* = 0.040), maximum IPH area percentage (β, −0.127; 95% CI, from −0.223 to −0.030; *p* = 0.012), and ulcer (β, 0.069; 95% CI, 0.025–0.113; *p* = 0.005); rCBF_post−CEA_ was associated with the IPH volume (β, −0.060; 95% CI, from −0.107 to −0.014; *p* = 0.013). After adjusting for the confounding factors, the associations of calcification with rCBF_pre−CEA_ (β, 0.099; 95% CI, from 0.004 to −0.194; *p* = 0.042) and IPH volume with rCBF_post−CEA_ (β, −0.060; 95% CI, from −0.109 to −0.011; *p* = 0.020) remained statistically significant, while those of rCBF_pre−CEA_ with maximum IPH area percentage (β, −0.089; 95% CI, from −0.188 to 0.011; *p* = 0.080) and ulcer (β, 0.050; 95% CI, from −0.012 to 0.112; *p* = 0.100) did not remain statistically significant.

**Conclusion:**

The compositional characteristics of carotid atherosclerotic plaques, particularly IPH, were associated with perioperative CBF in patients with unilateral moderate-to-severe carotid stenosis undergoing CEA. Our findings indicated that the patients with larger carotid IPH could expect smaller improvement in CBF following CEA.

## Introduction

Stroke, characterized by high morbidity, disability, and mortality (1, 2), is one of the major causes of death worldwide. Carotid atherosclerosis stenosis accounts for 25–30% of adult strokes. The previous studies reported the reduction and redistribution of cerebral perfusion in patients with carotid stenosis and asymptomatic patients with subclinical cerebrovascular atherosclerosis (3, 4). Carotid endarterectomy (CEA) is a key intervention for moderate-to-severe carotid stenosis through which plaque removal, recanalization, and improved cerebral perfusion reduce the risk of a future stroke (5–7). Cerebral blood flow (CBF), a major physiological parameter of cerebral perfusion, usually changes before clinical symptoms appearing in patients with cerebrovascular disease (8–10). Therefore, an early description of the changes in CBF could help prevent ischemic events and evaluate the effects of vascular interventions.

Carotid atherosclerotic stenosis directly leads to reduction of ipsilateral CBF (11). Furthermore, disruption of the vulnerable carotid plaque characterized by intraplaque hemorrhage (IPH) or ulcer contributes to cerebral microcirculation obstruction by microemboli (12, 13). The previous studies demonstrated that perioperative CBF in patients with moderate-to-severe carotid stenosis was associated with plaque burden and components. Jongen et al. (14) found that carotid plaque burden and degree of stenosis were negatively associated with ipsilateral CBF as measured by computed tomography (CT) perfusion imaging in patients with symptomatic carotid stenosis (stenosis ≥50%) at baseline. A clinical study of 72 patients with carotid stenosis demonstrated that carotid IPH and calcification were significantly associated with baseline CBF and cerebrovascular reactivity which were determined by Xe-CT (15). Investigators also determined the relationships between carotid plaque characteristics and post-CEA CBF. Lishmanov et al. (16) reported that the CBF and cerebral blood volume measured by single-photon emission CT increased after CEA in patients with carotid stenosis. Our previous study demonstrated that both plaque burden and lipid-rich necrotic core (LRNC) were correlated with changes in CT perfusion measurements following CEA (17) indicating that, carotid plaque characteristics could predict the CBF in patients with carotid stenosis undergoing CEA.

The CBF can be assessed using cerebral perfusion technologies, including CT perfusion, dynamic susceptibility contrast magnetic resonance (MR) perfusion, and arterial spin labeling (ASL)-based perfusion imaging. However, CT imaging that emits ionizing radiation is not an ideal methodology for a repeated monitoring of cerebral perfusion changes perioperatively. Dynamic susceptibility contrast MR perfusion imaging is contraindicated for CBF assessment in patients with renal dysfunction. Arterial spin labeling (ASL) MR imaging (MRI) has good reproducibility and permits non-invasive quantification of blood flow using the protons of arterial blood water molecules as endogenous diffusible tracers (18–20). A study by Han et al. showed a reduced CBF in asymptomatic subjects with cerebrovascular atherosclerosis assessed by pseudo-continuous arterial spin labeling (pcASL) MRI (3). A clinical study of 19 patients with unilateral internal carotid atherosclerotic stenosis undergoing CEA demonstrated the effectiveness of ASL MRI in predicting the surgical hemodynamic outcomes (21). Other investigators reported the characteristics of territorial CBF distribution in patients undergoing carotid stenting and CEA assessed by ASL MRI (22). However, few studies utilized ASL MRI to determine the associations between carotid plaque characteristics and perioperative CBF in patients undergoing CEA.

This study aimed to evaluate the associations between the carotid plaque characteristics and CBF in patients with unilateral moderate-to-severe carotid stenosis before and after CEA, using carotid vessel wall and brain ASL MRI. This study could help find a surrogate indicator of CBF for perioperative brain perfusion in patients undergoing CEA.

## Materials and Methods

### Study Sample

Symptomatic and asymptomatic patients with moderate-to-severe unilateral carotid stenosis (50–99%) determined by CT angiography and referred for CEA were prospectively recruited and underwent brain and carotid artery MRI. The exclusion criteria included: (1) ≥50% stenosis in the contralateral internal carotid artery; (2) significant stenoses (≥50%) or occlusions in the intracranial vasculature; (3) acute infarction; (4) cerebral tumors; (5) underwent a vascular intervention treatment such as CEA or carotid stenting; (6) contraindications to MRI examination. Clinical information (e.g., age, sex, and history of hypertension, diabetes, hyperlipidemia, smoking, and drinking) was collected from the clinical records. This study was approved by the local ethic committee. The study was performed in accordance with the ethical standards laid down in the 1964 Declaration of Helsinki and its later amendments. All enrolled patients signed written informed consent.

### Carotid Artery and Brain MRI

The carotid vessel wall MRI was performed on a 3.0 T scanner (uMR780, United Imaging Healthcare, Shanghai, China) with an 8-channel carotid coil within 1 week before the CEA. The multi-contrast MR vessel wall imaging protocol consisted of (1) 3D time-of-flight: gradient echo; repetition time (TR)/echo time (TE), 17.6/6.7 ms; flip angle, 8°; slice thickness, 2 mm. (2) 2D T1-MATRIX (modulated flip angle technique in refocused imaging with extended echo train): fast spin echo (FSE); TR/TE, 850/13.44 ms; slice thickness, 2 mm. (3) 2D T2-MATRIX: FSE; TR/TE, 2,000/96.6 ms; slice thickness, 2 mm. (4) Simultaneous non-contrast angiography intraplaque hemorrhage sequence: gradient echo; TR/TE, 9.6/4.0 ms; flip angle, 12°; slice thickness, 1 mm. The field of view, spatial resolution, and longitudinal coverage were 140 × 140 mm^2^, 0.55 × 0.55 mm^2^, and 32 mm, respectively. The MR scanning was centered on the index carotid artery, defined as the artery with moderate-to-severe stenosis referred for CEA.

All patients also underwent brain MRI on a 3.0 T scanner (Discovery 750, GE Medical Systems) equipped with an 8-channel head coil within 1 week before and 72 h after the CEA. The conventional imaging sequences and parameters were as follows: (1) T1-weighted imaging: FSE; TR/TE, 2,374/24 ms; matrix size, 352 × 256. (2) T2-weighted imaging: FSE; TR/TE, 9,266/73 ms; matrix size, 384 × 384. (3) Fluid-attenuated inversion recovery: FSE; TR/TE, 8,400/140 ms; matrix size, 284 × 224. (4) Diffusion-weighted imaging: echo-planar imaging; TR/TE, 3,000/71 ms; matrix size, 160 × 160; b = 0 and b = 1,000. All sequences were acquired with the same field of view of 250 × 250 mm^2^ and slice thickness of 5 mm. In addition, a 3D-pcASL sequence was acquired for the entire brain with the following parameters: TR/TE, 4,632/10.5 ms; slice thickness, 4 mm; slice interval, 0; voxel size, 2 × 2 × 4 mm^3^; post-labeling delay, 2,000 ms; scanning time, 3 min 45 s.

### Surgical Procedures

All CEA procedures were conducted by the same senior neurosurgeon with over 30 years of experience, following standardized surgical procedures under general anesthesia. Cerebral oxygen saturation and microembolism were monitored throughout the perioperative period. Deep neck dissection and vessel manipulation were performed under a microscope. The plaques and thickened intima were carefully removed to achieve a smooth vessel wall.

### Data Analysis

The carotid vessel wall MR images were reviewed by two trained observers with >2 years of experience in cerebrovascular imaging using custom-designed software (Vessel Explorer 2, TSimaging Healthcare, Beijing, China). When the interpretation results were inconsistent between these two observers, a senior observer with more than 10 years of experience reviewed the images. All three observers were blinded to the clinical information and status of the cerebral collateral circulation. The image quality was assessed using a 4-point scale (1 = poor, 2 = marginal, 3 = good, and 4 = excellent). Only those with image quality ≥ 2 were included for further analysis. The plaque morphology assessment included: mean lumen, wall, and total vessel areas; maximum wall thickness; and normalized wall index [(%) = wall area/total vessel area ×100]. These were measured by manually outlining the lumen and wall boundaries for each index artery. The presence of carotid plaque components, including calcification, LRNC, IPH, ulcer, and fibrous cap rupture, was assessed using published criteria (23). The volume and maximum area percentage of plaque components, including calcification, LRNC, and IPH, were measured for each patient. Excellent inter-observer and intra-observer agreements were found in carotid plaque morphology evaluations (17).

The whole-brain 3D-pcASL MRI scans were transferred to an MR workstation (AW 4.6, GE Healthcare, Waukesha, WI, USA) for post-processing. Two observers with more than 2 years of experience in processing ASL images drew the regions of interest corresponding to the cortical flow territory of the middle cerebral artery on the index and contralateral sides of each axial slice. The observers were blinded to the degree of carotid stenosis, carotid vessel wall images, and clinical information. Three slices, one at the level of the basal ganglia and the adjacent up and down slices, were used to eliminate changes in the CBF value in the middle cerebral artery territory. The regions of obsolete cerebral infarction within the regions of interest were excluded from the CBF calculations. The average absolute CBF value was calculated from the CBF measurement of the three slices on each side. The relative CBF (rCBF) was calculated by dividing the absolute CBF value on the index side by the absolute CBF value on the contralateral side. The rCBF values were calculated before and after the CEA procedure, respectively.

### Statistical Analysis

Continuous variables with normal distribution are presented as means with standard deviations, and those with abnormal distribution are described as medians with interquartile ranges. The correlation between rCBF and the clinical information was analyzed using the Pearson's or Spearman's correlation coefficients. The correlations between the plaque characteristics and rCBF were also analyzed by univariate and multi-variate linear regressions before and after adjusting for confounding factors. Two-tailed *p* < 0.05 was considered statistically significant. Statistical analysis was performed using IBM SPSS Statistics for Windows, Version 19.0 (IBM Corp., Armonk, NY, USA).

## Results

### Clinical and Carotid Plaque Characteristics

This study enrolled 66 patients, of which 5 patients were excluded because of the missing postoperative whole-brain 3D-pcASL MRI (*n* = 3) or poor vessel wall MRI quality due to the motion artifacts (*n* = 2). Of the remaining 61 patients (mean age, 66.6 ± 7.8 years), 55 (90.2%) are males, 41 (67.2%) had hypertension, 33 (54.1%) had hyperlipidemia, 23 (37.7%) had diabetes, 40 (65.6%) were smokers (present or past), and 12 (30.8%) were drinkers (present or past). The patient and clinical characteristics are summarized in Table 1.

**Table 1:** Clinical characteristic of study population (*n* = 61).

	**Mean ± SD, *n* (%)**	**Range**
**Age**, years	66.6 ± 7.8	52–85
**Sex**, male	55 (90.2)	-
**Hypertension**	41 (67.2)	-
SBP, mm Hg	132.1 ± 16.4	91–200
DBP, mm Hg	80.3 ± 9.0	60–100
**Hyperlipidemia**	33 (54.1)	-
HDL, mmol/L	1.1 ± 0.3	0.7–2.0
LDL, mmol/L	2.2 ± 0.8	0.9–4.2
TC, mmol/L	3.7 ± 0.9	2.4–6.1
TG, mmol/L	1.4 ± 0.7	0.4–3.1
**Diabetes**	23 (37.7)	-
Glu, mmol/L	6.1 ± 1.5	4.2–11.3
**Smoking history**	37 (60.7)	-
**Alcohol history**	40 (65.6)	-

*SBP, systolic blood pressure; DBP, diastolic blood pressure; HDL, high density lipoprotein; LDL, low density lipoprotein; TC, total cholesterol; TG, triglyceride; Glu, glucose. The categorical variables were described as percentage and continuous variables were described as median and interquartile range*.

Among the 61 patients, 52 (85.2%), 28 (45.9%), 48 (78.7%), 15 (24.6%), and 32 (52.5%) had carotid LRNC, IPH, calcification, ulcer, and fibrous cap rupture, respectively. Other morphological and component characteristics are shown in Table 2.

**Table 2:** Carotid plaque characteristics of study population (*n* = 61).

	**Median (IQR), *n* (%)**
**Morphology**	
Mean lumen area, mm^2^	26.1 (21.1, 32.6)
Mean wall area, mm^2^	41.6 (31.6, 56.2)
Mean total vessel area, mm^2^	67.4 (57.5, 87.6)
Maximum wall thickness, mm	6.0 (4.8, 7.3)
Mean normalized wall index, %	61.8 (55.8, 66.0)
Stenosis, %	74.5 (68.8, 82.1)
**Presence of plaque components**	
LRNC	52 (85.2)
IPH	28 (45.9)
Calcification	48 (78.7)
Ulcer	15 (24.6)
FCR	32 (52.5)
**Volume of plaque components** ^*^	
LRNC, mm^3^	416.6 (159.1, 789.7)
IPH, mm^3^	374.9 (159.9, 688.7)
Calcification, mm^3^	41.3 (14.5, 74.7)
Ulcer, mm^3^	19.3 (6.2, 44.3)
**Maximum area percentage of plaque components** ^*^	
LRNC, %	62.5 (38.1, 72.1)
IPH, %	50.7 (35.4, 61.4)
Calcification, %	8.8 (5.6, 16.6)
Ulcer, %	4.4 (3.3, 17.9)

*LRNC, lipid-rich necrotic core; IPH, intraplaque hemorrhage. All the measurements were described as median and interquartile range*.

* *Only patients with the corresponding component present were included in the comparison*.

### Correlations Between Clinical Information and CBF

**Figure 1:**
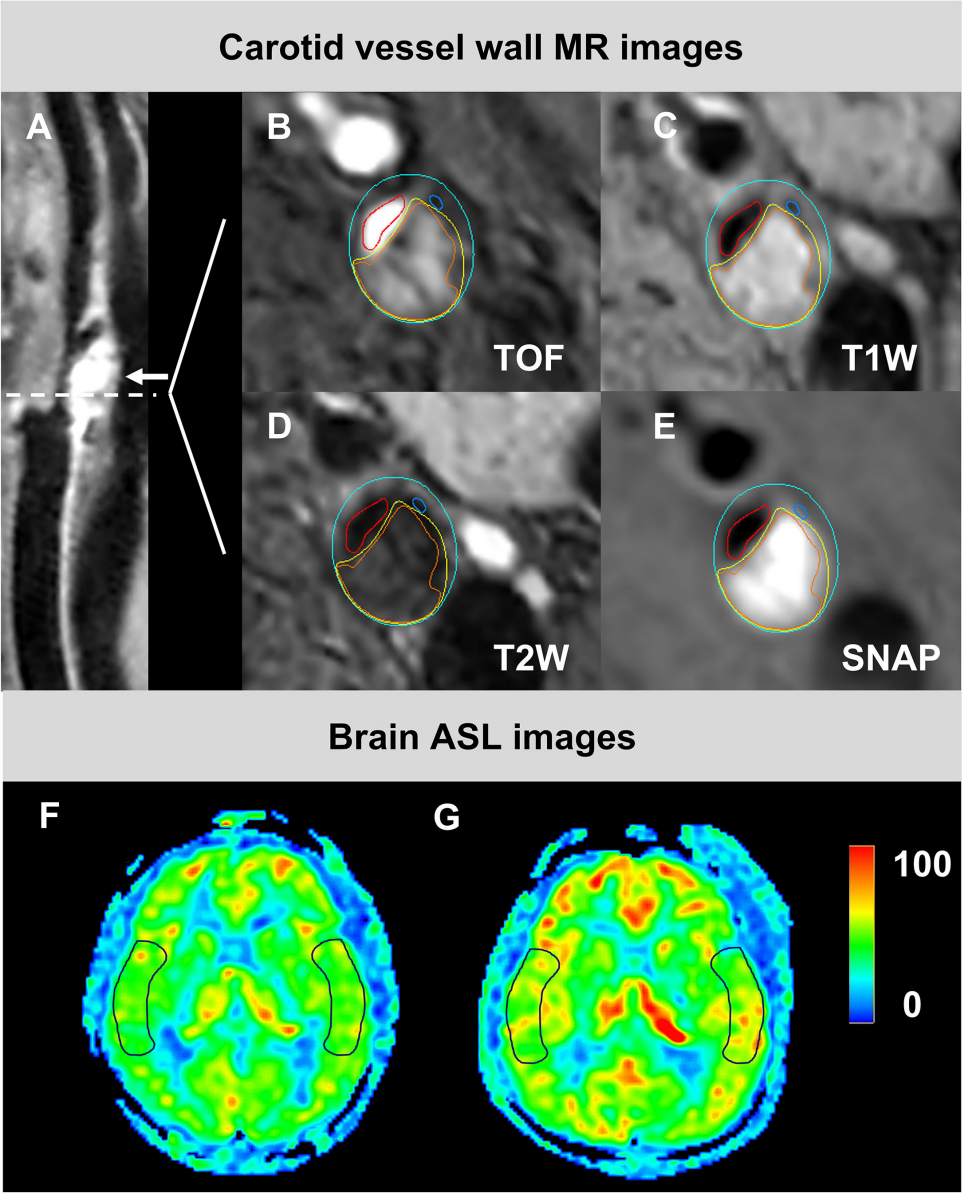
The images from a 85-year-old male patient who had carotid atherosclerotic plaque with IPH and calcification in the left side and changed CBF undergoing CEA. An eccentric plaque (white arrow) was delineated in the curved projection reconstructed image of 3D T1W **(A)**. IPH (orange), calcification (navy blue), and LRNC (yellow) were demonstrated on the axial multi-contrast MR images with the manually outlied lumen (red), wall (blue), and component boundaries **(B–E)**. The preoperative **(F)** and postoperative **(G)** CBF maps were shown, respectively. The ROIs on images **(F,G)** were located at the cortical flow territory of the middle cerebral artery on both index and contralateral sides. CBF, cerebral blood flow; CEA, carotid endarterectomy; IPH, intraplaque hemorrhage; LRNC, lipid-rich necrotic core; ROIs, region of interests; SNAP, Simultaneous non-contrast angiography intraplaque hemorrhage; T1W, T1-weighted; T2W, T2-weighted. TOF, time-of-flight.

The associations of the clinical risk factors with CBF_pre−CEA_ and CBF_post−CEA_ (Figure 1) on the index side are summarized in Table 3. A significant correlation was found between diastolic blood pressure and CBF_pre−CEA_ (*r* = −0.279, *p* = 0.029). No significant correlations were found between other clinical factors and CBF measurements (all *p* > 0.05).

**Table 3:** Correlations between clinical information and CBF (*n* = 61).

	**rCBF**_**pre−CEA**_		**rCBF**_**post−CEA**_	
	***r***	***p***	***r***	***p***
**Age**, years	−0.005	0.972	0.188	0.147
**Gender**, male	0.100	0.443	0.109	0.401
**Hypertension**	−0.083	0.523	−0.143	0.272
SBP, mmHg	0.035	0.789	−0.065	0.619
DBP, mmHg	−0.279	0.029	−0.092	0.479
**Hyperlipidemia**	−0.032	0.808	−0.051	0.694
HDL, mmol/L	0.059	0.650	0.016	0.906
LDL, mmol/L	0.140	0.282	−0.137	0.292
TC, mmol/L	0.157	0.228	−0.185	0.154
TG, mmol/L	0.008	0.954	−0.225	0.081
**Diabetes**	−0.086	0.508	0.044	0.735
Glu, mmol/L	0.060	0.646	0.101	0.438
**Smoking history**	0.162	0.212	0.081	0.535
**Alcohol history**	−0.014	0.916	−0.052	0.691

*SBP, systolic blood pressure; DBP, diastolic blood pressure; HDL, high density lipoprotein; LDL, low density lipoprotein; TC, total cholesterol; TG, triglyceride; Glu, glucose. The categorical variables were described as percentage and continuous variables were described as median and interquartile range*.

### Correlations Between the Carotid Plaque Characteristics and CBF

The associations of the carotid plaque characteristics with CBF_pre−CEA_ and CBF_post−CEA_ are shown in Tables 4, 5. A significant negative correlation was found between carotid stenosis and CBF_pre−CEA_ (β, −0.462; 95% CI, from −0.797 to −0.126; *p* = 0.008). No significant correlations were found between other morphological characteristics and CBF (all *p* > 0.05).

**Table 4:** Univariate linear regression analysis between plaque characteristics and CBF (*n* = 61).

	**rCBF**_**pre−CEA**_			**rCBF**_**post−CEA**_		
	**β**	**95% CI**	***p***	**β**	**95% CI**	***p***
**Morphology**						
Mean lumen area, mm^2^	−0.003	−0.007, 0.001	0.194	−0.001	−0.005, 0.002	0.375
Mean wall area, mm^2^	−0.001	−0.003, 0.001	0.278	−0.001	−0.003, 0.001	0.314
Mean total vessel area, mm^2^	−0.001	−0.003, 0.001	0.178	−0.001	−0.002, 0.001	0.260
Max WT, mm	−0.009	−0.031, 0.013	0.398	−0.014	−0.031, 0.003	0.099
Mean NWI, %	0.001	−0.004, 0.005	0.819	0.002	−0.002, 0.006	0.300
Stenosis, %	−0.462	−0.797, −0.126	0.008	0.057	−0.221, 0.336	0.683
**Presence of plaque components**					
LRNC	−0.018	−0.135, 0.100	0.762	0.028	−0.064, 0.119	0.549
IPH	−0.016	−0.099, 0.068	0.708	0.053	−0.011, 0.117	0.101
Calcification	0.103	0.005, 0.201	0.040	−0.014	−0.094, 0.065	0.722
Ulcer	0.083	−0.011, 0.177	0.084	−0.025	−0.100, 0.051	0.515
FCR	0.020	−0.063, 0.103	0.631	0.044	−0.020, 0.108	0.176
**Volume of plaque components** ^*^ ^**#**^					
LRNC, mm^3^	−0.029	−0.077, 0.020	0.240	−0.030	−0.065, 0.006	0.098
IPH, mm^3^	−0.045	−0.114, 0.023	0.182	−0.060	−0.107, −0.014	0.013
Calcification, mm^3^	0.022	−0.019, 0.063	0.286	−0.004	−0.033, 0.025	0.805
Ulcer, mm^3^	0.041	−0.011, 0.093	0.112	0.013	−0.023, 0.049	0.454
**Maximum area percentage of plaque components** ^*^ ^**#**^						
LRNC, %	−0.029	−0.091, 0.033	0.355	−0.001	−0.048, 0.046	0.965
IPH, %	−0.127	−0.223, −0.030	0.012	−0.026	−0.106, 0.055	0.515
Calcification, %	0.026	−0.019, 0.070	0.251	0.012	−0.019, 0.043	0.441
Ulcer, %	0.069	0.025, 0.113	0.005	0.025	−0.010, 0.061	0.146

* *Only patients with the corresponding component present were included in the comparison*.

# *The increment of these continuous variables was 1 standard deviation. LA, lumen area; WA, wall area; TVA, total vessel area; Max WT, maximum wall thickness; NWI, normalized wall index; LRNC, lipid-rich necrotic core; IPH, intraplaque hemorrhage; and FCR, fibrous cap rupture*.

**Table 5:** Multi-variate linear regression analysis between plaque characteristics and CBF (*n* = 61).

	^*^ **rCBF**_**pre−CEA**_			^**^ **rCBF**_**post−CEA**_		
	**β**	**95% CI**	***p***	***B***	**95% CI**	***p***
**Morphology**						
Mean lumen area, mm^2^	−0.001	−0.005, 0.003	0.548	−0.002	−0.005, 0.001	0.300
Mean wall area, mm^2^	−0.0004	−0.003, 0.002	0.725	−0.0009	−0.003, 0.001	0.311
Mean total vessel area, mm^2^	−0.0004	−0.002, 0.001	0.607	−0.0008	−0.002, 0.001	0.236
Mas WT, mm	−0.003	−0.024, 0.018	0.797	−0.011	−0.028, 0.006	0.193
Mean NWI, %	0.002	−0.003, 0.007	0.373	0.002	−0.003, 0.006	0.415
**Presence of plaque components**						
LRNC	0.008	−0.111, 0.128	0.888	0.022	−0.077, 0.121	0.659
IPH	−0.022	−0.109, 0.064	0.606	0.051	−0.020, 0.121	0.154
Calcification	0.099	0.004, 0.194	0.042	−0.055	−0.139, 0.030	0.200
Ulcer	0.054	−0.037, 0.146	0.238	−0.012	−0.089, 0.065	0.749
FCR	0.016	−0.065, 0.098	0.689	0.033	−0.034, 0.100	0.327
**Volume of plaque components** ^#^ ^†^						
LRNC, mm^3^	−0.026	−0.070, 0.019	0.260	−0.024	−0.060, 0.011	0.174
IPH, mm^3^	−0.038	−0.099, 0.023	0.213	−0.060	−0.109, −0.011	0.020
Calcification, mm^3^	0.022	−0.019, 0.062	0.285	−0.007	−0.037, 0.022	0.618
Ulcer, mm^3^	0.026	−0.031, 0.083	0.329	0.021	−0.024, 0.065	0.322
**Maximum area percentage of plaque components** ^#^ ^†^						
LRNC, %	−0.017	−0.076, 0.042	0.559	0.002	−0.044, 0.048	0.933
IPH, %	−0.089	−0.188, 0.011	0.080	0.040	−0.130, 0.051	0.371
Calcification, %	0.026	−0.018, 0.071	0.233	0.011	−0.021, 0.043	0.497
Ulcer, mm^3^	0.050	−0.012, 0.112	0.100	0.038	−0.004, 0.081	0.072

* *Adjusted for age, sex, index-stenosis and DBP;*

** *Adjusted for age, sex, index-stenosis and TG;*

# *Only patients with the corresponding component present were included in the comparison.*

† *The increment of these continuous variables was 1 standard deviation. Max WT, maximum wall thickness; NWI, normalized wall index; LRNC, lipid-rich necrotic core; IPH, intraplaque hemorrhage; FCR, fibrous cap rupture*.

The univariate linear regression analysis showed a significant association between calcification and CBF_pre−CEA_ (β, 0.103; 95% CI, 0.005–0.201; *p* = 0.040). After adjusting for age, sex, index-stenosis, and DBP, this association remained statistically significant (β, 0.099; 95% CI, from 0.004 to −0.194; *p* = 0.042). No other plaque components were found to be associated with any of the CBF measurements before or after adjusting for the confounding factors (all *p* > 0.05). The univariate linear regression analysis showed significant associations between CBF measurements and the IPH size (maximum IPH area percentage with rCBF_pre−CEA_: β, −0.127; 95% CI, from −0.223 to −0.030; *p* = 0.012; maximum ulcer area percentage with rCBF_pre−CEA_: β, 0.069; 95% CI, 0.025–0.113; *p* = 0.005; IPH volume with rCBF_post−CEA_: β, −0.060; 95% CI, from −0.107 to −0.014; *p* = 0.013). After adjusting for the confounding factors, the association between IPH volume and rCBF_post−CEA_ remained statistically significant (β, −0.060; 95% CI, from −0.109 to −0.011; *p* = 0.020), but the associations between rCBF_pre−CEA_ and the maximum IPH area percentage (β, −0.089; 95% CI, from −0.188 to 0.011; *p* = 0.080) and ulcer (β, 0.050; 95% CI. −0.012 to 0.112; *P* = 0.100) did not remain statistically significant. No significant correlations were found between other plaque size measurements and any of the CBF measurements before and after adjusting for the confounding factors (all *p* > 0.05).

## Discussion

This study investigated the associations between the carotid plaque characteristics and the perioperative CBF in patients with unilateral moderate-to-severe stenosis using multi-contrast carotid vessel wall and whole-brain 3D-pcASL MRI. We found that calcification was positively correlated with CBF before CEA, and the IPH size was negatively correlated with CBF after CEA. The data indicated that the patients with calcification had higher CBF at baseline than those without, and patients with large carotid IPH achieved lower CBF following CEA than those with small or no carotid IPH.

We found that the stenosis of index carotid artery was negatively correlated with the CBF at baseline, consistent with the previous studies (14, 24). Many studies demonstrated that the CBF decreased with the increase of the carotid stenosis degree (14, 24). Tang et al. (24) reported that the carotid artery blood flow on the index side decreased from 418.4 ml/min to 301.0 ml/min, with stenosis of index carotid artery increasing from no stenosis to severe stenosis. Carotid artery stenosis will directly lead to hypoperfusion in the downstream cerebral tissues if the collateral circulation is insufficient. Furthermore, the investigators found that the atherosclerotic plaque vulnerability was significantly associated with the degree of carotid stenosis. Zhao et al. (25) reported that the prevalence of fibrous cap rupture was 23.2%, 33.3%, and 53.8% in carotid arteries with 1–49%, 50–69%, and ≥70% stenosis, respectively. The European Carotid Surgery Trial included 3,007 patients with symptomatic carotid stenosis, reported that the prevalence of plaque surface irregularity (35–65%) and thrombus formation (20–45%) increased with the increase of degree of carotid stenosis from 10 to 99% (4). Fibrous cap rupture or surface irregularity in carotid arteries stimulates thrombosis that might lead to embolism and cerebral hypoperfusion.

In the present study, we found a positive correlation between calcification and the CBF at baseline, consistent with previous studies (15). A clinical study of 72 patients with unilateral carotid stenosis reported that intraplaque calcification was positively correlated with cerebrovascular reactivity (*r*^2^ = 0.282, *p* = 0.016) (15). Our findings might have arisen from various compensatory mechanisms during carotid plaque progression. Atherosclerosis is a chronic inflammatory disease of the arteries. It starts with the internalization and deposition of lipids in the intima, followed by phagocytosis of the lipids by macrophages to form foam cells. Calcium deposition occurs at advanced stages. Calcification might be a marker of a long-term progression of the carotid plaque, yielding blood flow compensation in the downstream tissues through either the Circle of Willis or leptomeningeal collateral channels. These compensatory mechanisms partially accounted for the small impact of the reduced CBF at baseline. This study suggested that patients with a calcified carotid plaque may better compensate for the reduced CBF through collaterals than those without calcification.

This study revealed that the carotid IPH size was negatively correlated with CBF after CEA. We speculated that this finding might be attributed to the rapid carotid plaque progression caused by IPH and the subsequent downstream micro-disruption. Several studies suggested that IPH was a trigger of plaque vulnerability and a powerful indicator of plaque progression (26–30). Histopathological studies suggested that IPH was primarily related to the extravasation of erythrocytes and leucocytes from immature micro-vessels in the adventitia (26, 31). An autopsy study showed that IPH might be a potent atherogenic stimulus by contributing to macrophage infiltration, free cholesterol deposition, and enlargement of the lipid-rich necrotic core (27). Another longitudinal *in vivo* study suggested that carotid IPH accelerated plaque progression over an 18-month follow-up period (28). Impaired microcirculation in patients with carotid stenosis and a larger-sized vulnerable IPH plaque might account for the diminished improvement in CBF after removing the plaque (12, 13). Our findings suggested that the patients with large-sized carotid IPH might experience a smaller improvement in the CBF after revascularization than those with smaller carotid IPH.

This study had several limitations. First, its small sample warrants further large-sample studies. Second, the collateral circulation status, particularly in the Circle of Willis, was not analyzed. Such collateral circulation could affect the perioperative CBF. Third, we only used one post-labeling delay (2,000 ms) during the ASL imaging, which may have led to an underestimation of the CBF in patients with slow blood flow due to severe carotid stenosis. Future studies using multiple delay times during ASL imaging are suggested. Finally, this study evaluated the CBF for only 72 h after CEA. Long-term CBF improvement after CEA needs further investigation.

In conclusion, compositional characteristics of carotid atherosclerotic plaques, particularly IPH, were associated with perioperative CBF in patients with unilateral moderate-to-severe carotid stenosis undergoing CEA. Clinicians should focus on plaque calcification and IPH, in addition to the level of carotid stenosis. This study suggested that plaques with calcification were more stable, and patients with atherosclerotic carotid stenosis and large IPH might achieve smaller CBF improvement after CEA than those without.

## Data Availability Statement

The original contributions presented in the study are included in the article/supplementary material, further inquiries can be directed to the corresponding author/s.

## Ethics Statement

The studies involving human participants were reviewed and approved by the Medical Ethics Committee of Peking University Third Hospital. The patients/participants provided their written informed consent to participate in this study.

## Author Contributions

Material preparation and data collection and analysis were performed by YL, RH, HX, TW, and GZ. The first manuscript draft was written by YL. Manuscript revision was made by HY, XZ, RH, and YL. All authors contributed to the study conception and design. All authors commented on previous versions of the manuscript and approved the final one.

## Conflict of Interest

The authors declare that the research was conducted in the absence of any commercial or financial relationships that could be construed as a potential conflict of interest.

## Publisher's Note

All claims expressed in this article are solely those of the authors and do not necessarily represent those of their affiliated organizations, or those of the publisher, the editors and the reviewers. Any product that may be evaluated in this article, or claim that may be made by its manufacturer, is not guaranteed or endorsed by the publisher.
